# Activation of NADPH oxidases leads to DNA damage in esophageal cells

**DOI:** 10.1038/s41598-017-09620-4

**Published:** 2017-08-30

**Authors:** Vikas Bhardwaj, Ravindran Caspa Gokulan, Andela Horvat, Liudmila Yermalitskaya, Olga Korolkova, Kay M. Washington, Wael El-Rifai, Sergey I. Dikalov, Alexander I. Zaika

**Affiliations:** 1 Department of Veterans Affairs, Tennessee Valley Healthcare System, Nashville, Tennessee USA; 2 Department of Surgery, Vanderbilt University Medical Center and Vanderbilt-Ingram Cancer Center, Nashville, Tennessee USA; 3 Department of Cancer Biology, Vanderbilt University Medical Center and Vanderbilt-Ingram Cancer Center, Nashville, Tennessee USA; 4 Division of Clinical Pharmacology, Vanderbilt University Medical Center and Vanderbilt-Ingram Cancer Center, Nashville, Tennessee USA; 5 Department of Pathology, Microbiology and Immunology, Vanderbilt University Medical Center and Vanderbilt-Ingram Cancer Center, Nashville, Tennessee USA

## Abstract

Gastroesophageal reflux disease (GERD) is the strongest known risk factor for esophageal adenocarcinoma. In the center of tumorigenic events caused by GERD is repeated damage of esophageal tissues by the refluxate. In this study, we focused on a genotoxic aspect of exposure of esophageal cells to acidic bile reflux (BA/A). Analyzing cells generated from patients with Barrett’s esophagus and human esophageal specimens, we found that BA/A cause significant DNA damage that is mediated by reactive-oxygen species. ROS originate from mitochondria and NADPH oxidases. We specifically identified NOX1 and NOX2 enzymes to be responsible for ROS generation. Inhibition of NOX2 and NOX1 with siRNA or chemical inhibitors significantly suppresses ROS production and DNA damage induced by BA/A. Mechanistically, our data showed that exposure of esophageal cells to acidic bile salts induces phosphorylation of the p47^phox^ subunit of NOX2 and its translocation to the cellular membrane. This process is mediated by protein kinase C, which is activated by BA/A. Taken together, our studies suggest that inhibition of ROS induced by reflux can be a useful strategy for preventing DNA damage and decreasing the risk of tumorigenic transformation caused by GERD.

## Introduction

Significant progress has been made in prevention and treatment of many human tumors during recent decades. Unfortunately, esophageal adenocarcinoma (EA) remains poorly treated, and the surgery that is the mainstay of current therapy carries notable morbidity and mortality. EA is also one of the fastest rising tumors in the US; its incidence has increased approximately 6-fold over the past 30 years.

The strongest known risk factor for EA is gastroesophageal reflux disease (GERD), which affects approximately 20% of the population in the US^1, 2^. Because of the disease, esophageal cells are exposed to a refluxate that comprises acidic content of the stomach frequently mixed with duodenal bile. A mix of gastric acid and bile causes significant tissue damage and induces inflammation, which in turn, exacerbates the mucosal injury. If damage persists, it can cause hyperplasia and Barrett’s esophagus (BE), a condition in which the normal squamous epithelial lining is replaced by a metaplastic intestinal type of epithelium. Although the origin of Barrett’s metaplasia remains a subject of ongoing debate, it is clear that further accumulation of genetic alterations in BE cells, induced by gastroesophageal reflux, may lead to esophageal dysplasia and EA. The molecular history, underlying this progression is poorly understood^3, 4^

DNA damage is a well-known factor that promotes tumor development. It is especially detrimental when damaged DNA is not fully repaired leading to generation of mutations. A number of studies including ours have found induction of reactive oxygen species (ROS) and DNA damage to follow the exposure to esophageal reflux. Animal experiments have also demonstrated that reflux, when it is experimentally induced, increases DNA damage, mutational rate and causes esophageal tumors recapitulating human pathology^5^. Previous studies have found that one of the important sources of ROS is NADPH oxidase NOX5-S, a truncated variant of NOX5. This protein was found to be involved in acid-induced generation of H_2_O_2_ and DNA damage^6–9^. Given the complex nature of ROS regulation, the aim of the present study was to investigate other mechanisms leading to induction of ROS and DNA damage by acidic bile salts.

## Results

### Acidic bile salts induce DNA damage in esophageal epithelial cells

**Figure 1:**
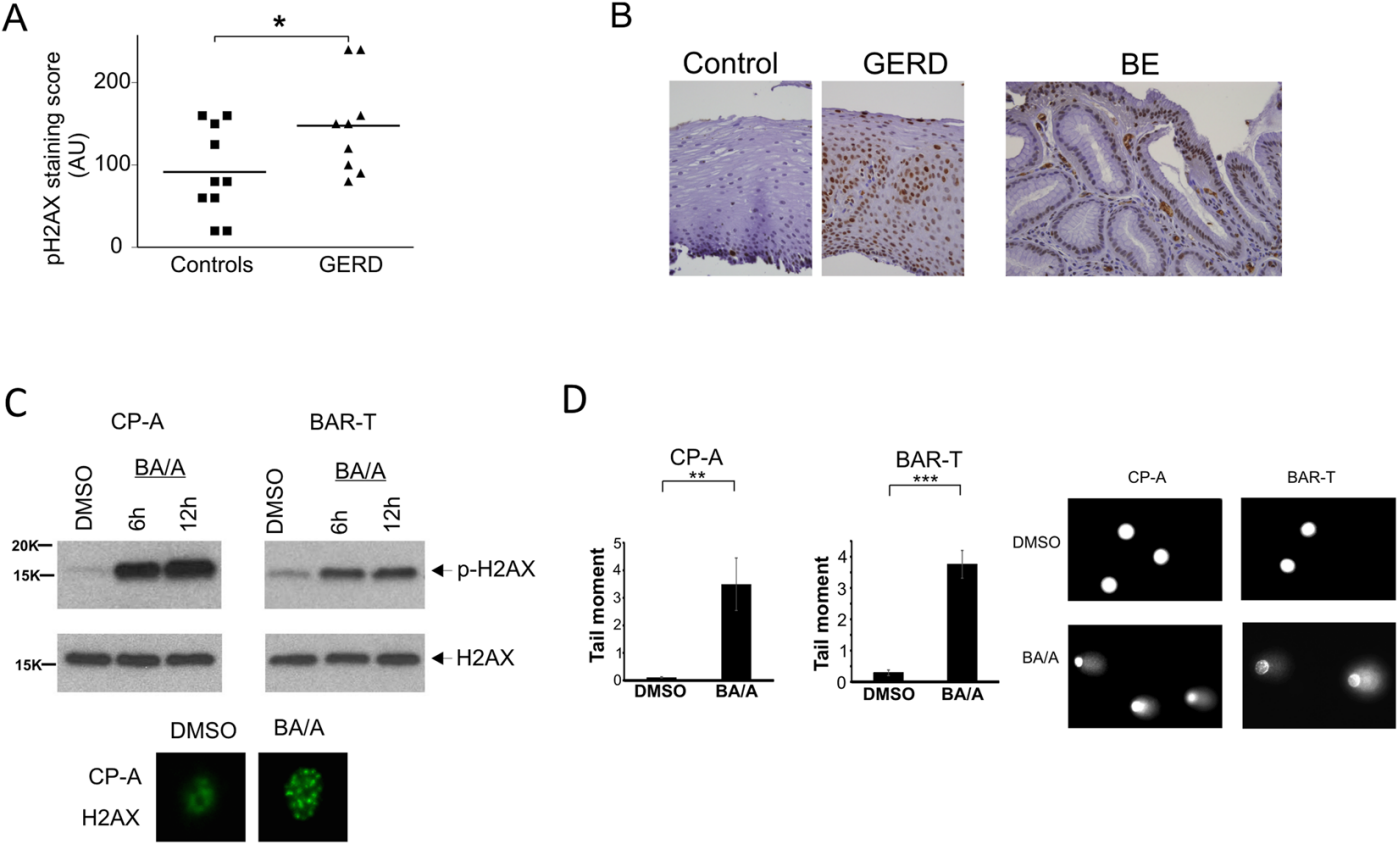
Esophageal reflux induces DNA damage in epithelial cells. (**A**) DNA damage was assessed in biopsies collected from GERD (n = 10) and control (n = 9) groups of patients using immunohistochemical staining for p-H2AX. Staining scores were calculated by multiplying the intensity score by the percentage of positively stained cells. GERD patients showed a statistically significant increase in p-H2AX staining compared to control patients without GERD (*p = 0.04, n = 19). (**B**) Representative images of p-H2AX staining of esophageal tissues collected from GERD, BE and control groups of patients. (**C**) Treatment with acidic bile salts induces DNA damage in CP-A and BAR-T cells. Top panel: CP-A and BAR-T cells were treated with BA/A (100 μM, pH 4.0) for 30 min and 5 min, respectively. Treated cells were analyzed for p-H2AX six and twelve hours after BA/A treatment using Western blotting. Bottom panel: A representative immunofluorescence staining for p-H2AX after treatment of CP-A cells with BA/A (100 μM, pH 4.0). Staining was done 12 hours after treatment. (**D**) Quantification of DNA damage by alkaline comet assay in CP-A and BAR-T cells treated with BA/A (100 μM, pH 4.0) for 30 min and 5 min, respectively. Analyses were conducted 18 hours after BA/A treatment (**p < 0.01, n = 3). Representative images of DNA comets are shown. Data are presented as mean ± S.E.

We started our study with analyses of DNA damage in GERD patients. Immunohistochemical staining for phosphorylated histone H2AX, a marker of DNA damage, was evaluated in 19 esophageal biopsies collected from GERD and control patients without GERD. We found a statistically significant increase (p = 0.04) in phospho-H2AX staining in esophageal epithelium collected from GERD patients compared to normal control group (Fig. 1A). DNA damage was also assessed in 10 biopsies collected from patients with Barrett’s esophagus (BE). We found that 4 out of 10 (40%) specimens have increased staining (staining intensity ≥2; Fig. 1B) for p-H2AX in Barrett’s epithelial cells, suggesting that DNA damage is increased in some BE patients.

To further investigate DNA damage in a more controlled environment, CP-A and BAR-T epithelial cells isolated from patients with BE, were exposed to acidic growth medium (pH 4.0) supplemented with 100 uM bile salt cocktail for the indicated time. The composition, total bile salts concentration and pH were selected based on previous measurements that allowed us to mimic a typical episode of reflux in GERD patients^10–12^. DNA damage in BA/A treated cells was assessed by Western blotting using phospho-H2AX antibody (Fig. 1C; bottom panels show *γ-*H2AX positive foci in CP-A cells treated with BA/A). Similar to GERD patients, we found that exposure to acidic bile salts causes DNA damage in both CP-A and BAR-T cells. We further confirmed these results using alkaline comet assay. We found significant increase in comet tail DNA content in BA/A-treated cells compared to untreated controls (Fig. 1D).

### Bile acids-associated DNA damage in esophageal epithelial cells is caused by ROS production

To elucidate the mechanism of DNA damage induced by GERD, we investigated ROS induction, since we and others have previously suggested a link between ROS and DNA damage induced by BA/A^8, 13–15^. We first selected a panel of antioxidants, which inhibit ROS by different mechanisms. The following compounds were selected: N-acetylсysteine (NAC), tempol, and apocynin. NAC has antioxidative and free radical scavenging properties through increasing intracellular GSH levels and its thiol-disulfide exchange activity^16^. Tempol is a strong membrane-permeable superoxide scavenger that exhibits a superoxide dismutase mimetic activity and confers catalase activity to heme proteins^17^. In contrast, apocynin possesses a relatively weak ROS scavenging activity but acts as an inhibitor of ROS-generating enzymes NADPH oxidases^18^.

**Figure 2:**
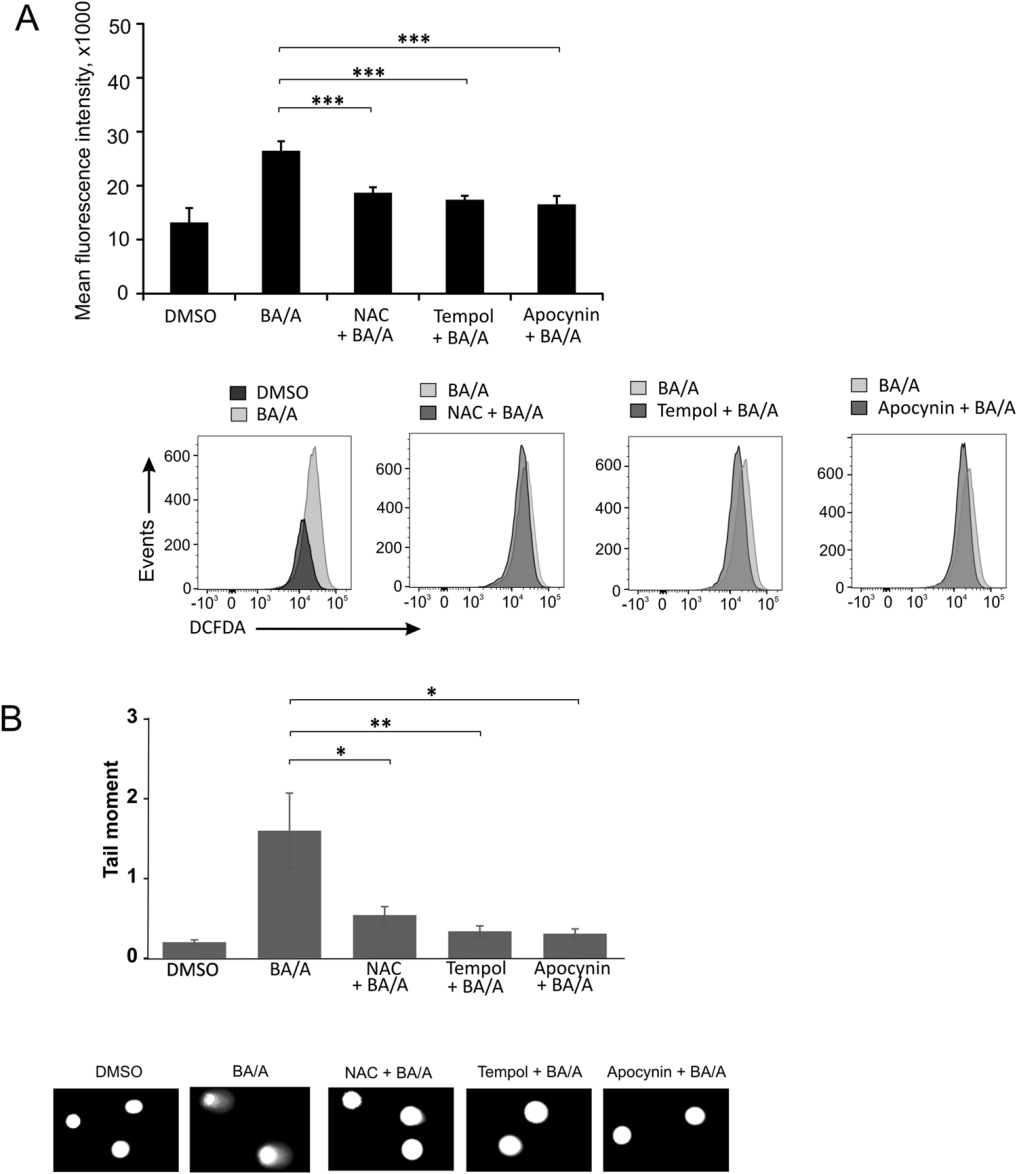
DNA damage induced by BA/A is mediated by ROS. CP-A cells were pre-treated with the indicated compounds (10 µM), and exposed to BA/A (100 μM, pH 4.0) for 30 min and analyzed 1 hour (**A**) and 18 hours (**B**) after BA/A treatment. (**A**) ROS were analyzed by flow cytometry using DCFDA staining. Representative flow cytometry profiles are shown. Graph shows mean fluorescence intensity ± S.E. (**p < 0.001, n = 3). (**B**) Analysis of DNA damage by comet assay. Representative images of DNA comets are shown. Data are presented as average ± S.E (*p < 0.05, n = 3).

CP-A cells were pre-treated with the aforementioned antioxidants for 1 hour. The treated cells were incubated with BA/A as described above, stained with H2DCFDA and then ROS levels were assessed using flow cytometry. We found that all tested compounds, despite different mechanisms of their antioxidant activity, were effective in reducing the ROS levels (Fig. 2A).

The effect of antioxidants on DNA damage was also assessed using comet assay (Fig. 2B). All tested antioxidants significantly suppressed DNA damage supporting the concept that ROS induction is an important cause of DNA damage induced by BA/A (Fig. 2B).

Finding inhibition of ROS by not only strong ROS scavengers, such as NAC and tempol, but also by NOX inhibitor apocynin suggested us that BA/A-generated ROS may originate from multiple sources.

**Figure 3:**
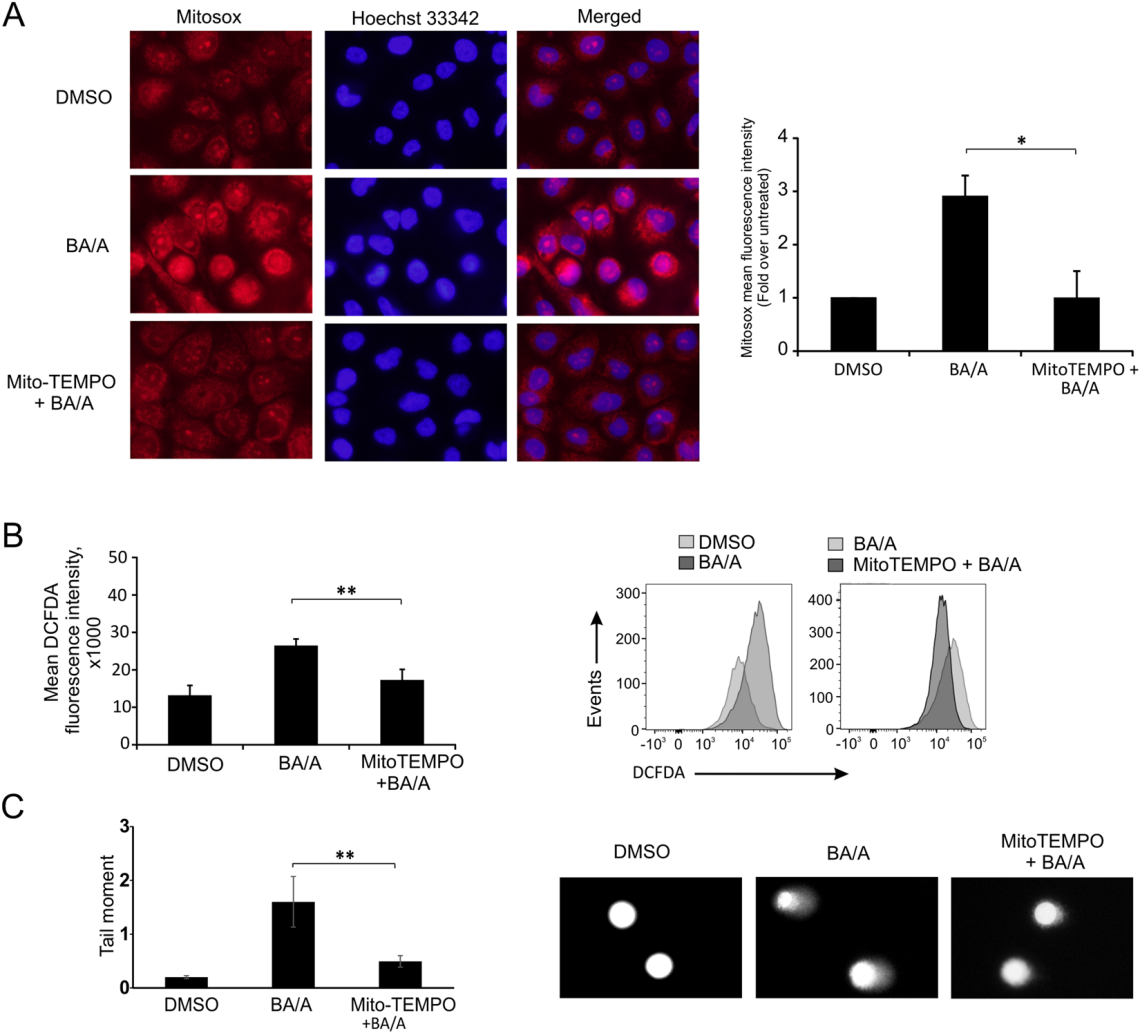
Treatment of esophageal cells with acidic bile salts (BA/A) increases mitochondrial ROS. CP-A cells were pre-treated with 0.1 μM MitoTEMPO or DMSO (diluent control), exposed to BA/A (100 μM, pH 4.0) for 30 min, and analyzed 1 hour (**A** and **B**) and 18 hours (**C**) after BA/A treatment. (**A**) Analysis of mitochondrial superoxide after BA/A treatment using MitoSOX Red. CP-A cells were stained with 5 µM MitoSOX Red (red signal) and 10 µM Hoechst 33342 (blue signal) to visualize the cell nuclei. The MitoSOX fluorescence was quantified at least in 50 cells for each treatment. Representative fluorescent images are shown on the left. Histogram shows the mean fluorescence intensity of MitoSOX from three independent experiments. Data are presented as mean ± S.E (*p = 0.02, n = 3). (**B**) ROS were analyzed by flow cytometry in CP-A cells using DCFDA staining. Representative flow cytometry profiles are shown. Histogram shows mean fluorescence intensity ± S.E. (**p < 0.001, n = 3). (**C**) Analyses of DNA damage in CP-A cells after treatment with the indicated compounds by comet assay. Representative images of DNA comets are shown. Data are presented as average ± S.E (**p < 0.01, n = 3).

To explore this possibility, we first analyzed the effect of acidic bile salts on mitochondrial ROS production. We used staining with MitoSOX Red, a fluorogenic dye that is highly selective for detection of mitochondrial superoxide. CP-A cells were treated with BA/A, stained with MitoSOX Red, and analyzed by fluorescent microscopy (Fig. 3A). Quantification of MitoSOX fluorescence showed significant increase in mitochondrial ROS production after exposure to BA/A (Fig. 3A). To confirm these data, we employed a specific mitochondrial ROS inhibitor MitoTEMPO, a mitochondria-targeted antioxidant with superoxide and alkyl radical scavenging properties^19^. Pre-treatment of CP-A cells with MitoTEMPO significantly decreased the ROS production induced by BA/A (Fig. 3A). The same conclusion was reached when ROS were analyzed using the DCFDA staining (Fig. 3B). In addition, inhibition of ROS by MitoTEMPO resulted in significant inhibition of DNA damage (Fig. 3C). Taken together, these data show that DNA damage in esophageal cells is mediated, at least in part, by generation of mitochondrial ROS caused by BA/A.

### NADPH oxidases are involved in generation of DNA damage by acidic bile salts

**Figure 4:**
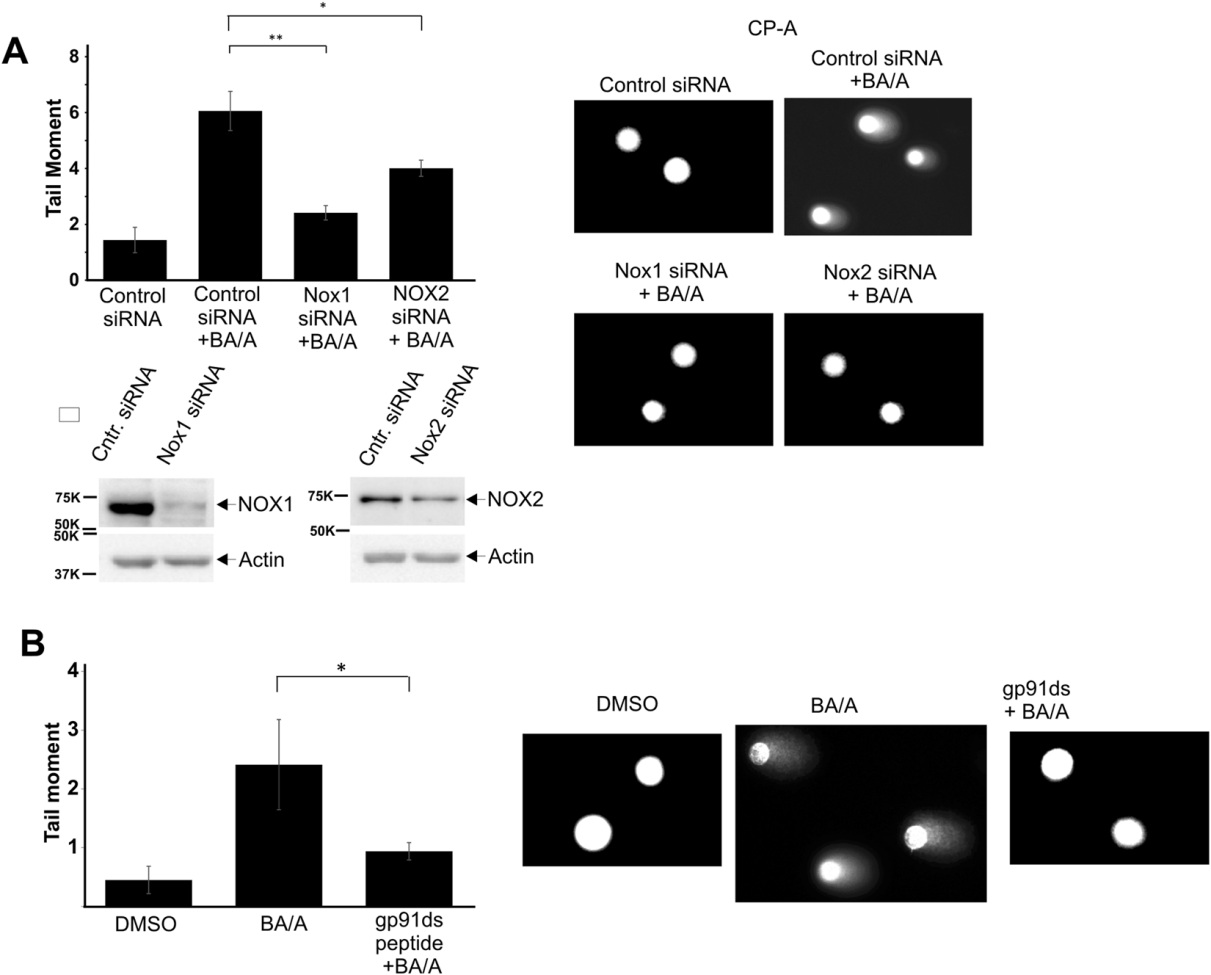
NOX1 and NOX2 are involved in generation of DNA damage after treatment with BA/A. (**A**) CP-A cells were transfected with siRNAs against NOX1, NOX2 or non-specific siRNA (control siRNA), exposed to BA/A (100 μM, pH 4.0), and analyzed for DNA damage using alkaline comet assay. Representative images of DNA comets are shown on the right. Data are presented as average ± S.E. (*p < 0.05, n = 3). Bottom panel shows protein expression of NOX1 and NOX2 analyzed by Western blotting. (**B**) CP-A cells were pre-treated with NOX2 inhibitory peptide gp91ds, exposed to BA/A (100 μM, pH 4.0), and analyzed for DNA damage using alkaline comet assay. Representative images of DNA comets are shown on the right. Data are presented as average ± S.E (*p < 0.05, n = 3).

Our screening of antioxidants identified apocynin as an effective inhibitor of ROS production and DNA damage caused by BA/A in Barrett’s cells (Fig. 2A and B (CP-A cells) and Supplementary Figure S1 (BAR-T cells)). Apocynin is considered to be a NOX inhibitor that affects the organizer NOX subunit p47^phox^, which is utilized by NOX2. It may also potentially affect NOX1, but not other NOX proteins. To examine the possibility that NOX2 and NOX1 are involved in generation of DNA damage by BA/A, these enzymes were downregulated using specific siRNAs in CP-A cells. Cells were then treated with BA/A for 30 minutes and analyzed for DNA damage by Comet assay. We found that DNA damage was significantly inhibited by either NOX1 or NOX2 downregulation, suggesting that both enzymes are involved in the generation of DNA damage (Fig. 4A). This finding was further supported by our experiments showing that a selective NADPH oxidase inhibitor - gp91ds peptide was also effective in inhibition of DNA damage (Fig. 4B).

**Figure 5:**
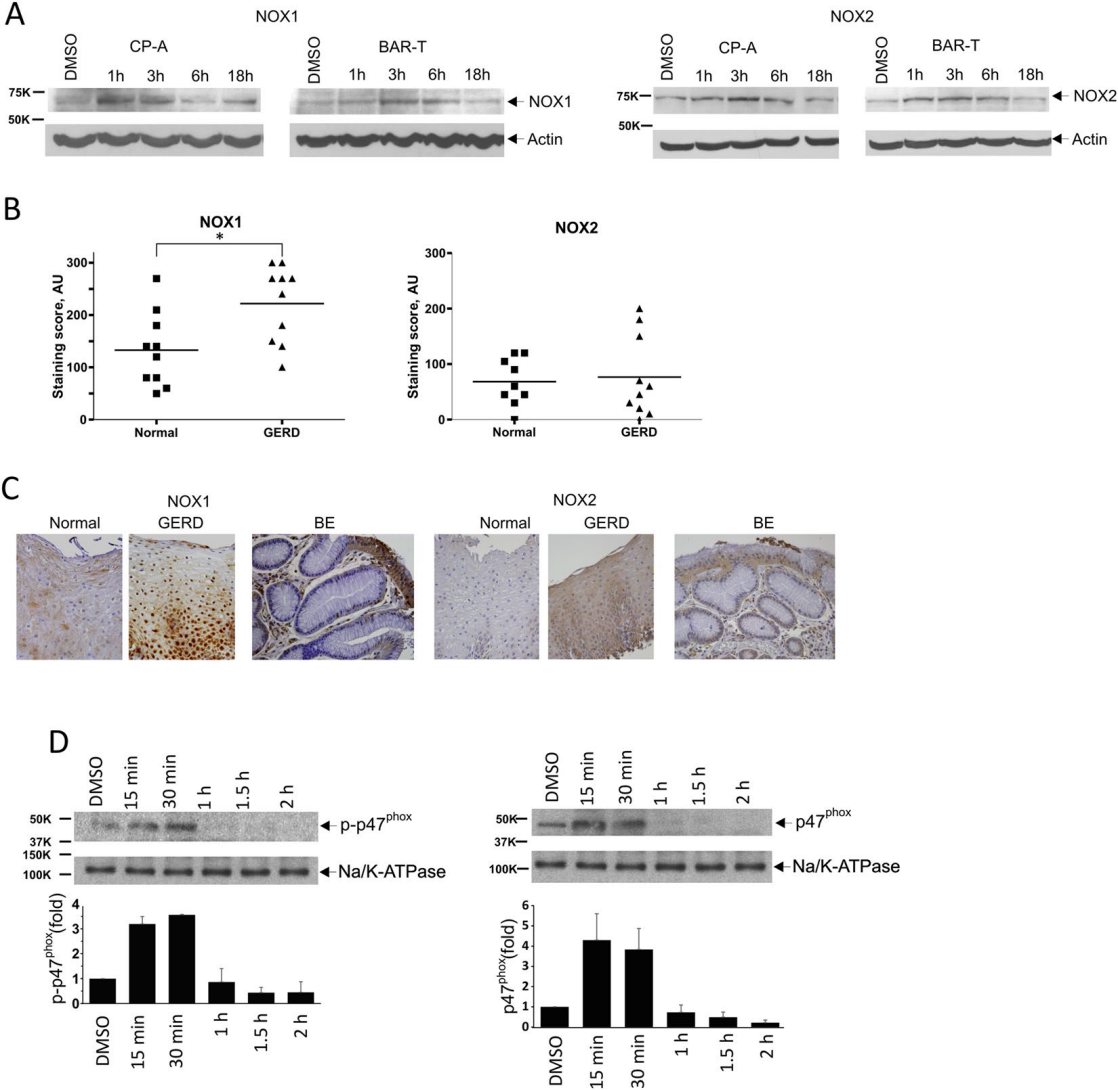
Exposure of esophageal cells to acidic bile salts leads to upregulation and activation of NAD(P)H oxidases. (**A**) CP-A and BAR-T cells were treated with BA/A (100 μM, pH 4.0) for 30 min and 5 min, respectively and analyzed at the indicated time using Western blotting with specific NOX1 and NOX2 antibodies. (**B**) Expression of NOX1 and NOX2 proteins were assessed in biopsies collected from GERD (n = 10) and control (n = 9) groups of patients using immunohistochemical staining with NOX1 and NOX2 antibodies. Staining scores were calculated by multiplying the intensity score by the percentage of positively stained cells. GERD patients showed a statistically significant increase in NOX1 expression compared to control patients (*p < 0.05, n = 19). NOX2 expression did not show statistically significant difference. (**C**) Representative images of NOX1 and NOX2 staining collected from GERD, BE and control groups of patients. (**D**) Treatment with BA/A induces phosphorylation of p47^phox^ subunit and its translocation to the cellular membrane. CP-A cells were treated with BA/A (100 μM, pH 4.0) and analyzed at the indicated time. Western blotting analyses were conducted in cellular membrane fraction. Na/K-ATPase served as a loading control. Expression of p47^phox^ protein was measured by densitometry after normalization to Na/K-ATPase. Levels of p47^phox^ protein in control cells were arbitrarily set at 1.

To analyze the regulation of NOX1 and NOX2, their expressions were assessed in CP-A and BAR-T cells treated with BA/A. We found a transient but strong increase in both NOX1 and NOX2 protein levels 1–3 hours after treatment (Fig. 5A). This increase has not occurred due to transcriptional upregulation, since no increase in levels of NOX1 or NOX2 mRNA were found in both CP-A and BAR-T cell lines, but rather due to post-translational mechanisms (Supplementary Figure S2).

To assess the expression of NOX proteins *in vivo*, 19 esophageal biopsies collected from GERD and normal patients were immunostained using NOX1 and NOX2 antibodies. Protein expression of NOX1 was significantly increased in esophageal epithelial and some inflammatory cells in GERD patients (Fig. 5B). In contrast, expression of NOX2 was highly varied among GERD patients; 33% (3 out of 9 patients) showed strong staining, while other patients did not show a significant difference from control group. We also assessed the protein expression of NOX1 and NOX2 in biopsies collected from BE patients. An increased staining (staining intensity ≥2; Fig. 5C) was found in 40% (4 out of 10 BE patients) for NOX1 and 20% (2 out of 10) for NOX2. Staining for NOX1 and NOX2 is shown in Fig. 5C.

These data show that exposure to BA/A *in vitro* and refluxate *in vivo* increases protein levels of NOX1 and NOX2 enzymes.

### NADPH oxidases are activated by acidic bile salts

Activation of NOX2 enzyme involves phosphorylation of p47^phox^ subunit and translocation of the NOX complex from the cytosol to the cellular membrane^20^. Based on a number of studies both *in vitro* and *in vivo*, p47^phox^ may also be involved in the regulation of NOX1^21–23^.

To investigate whether NOX2 and NOX1 enzymes are activated by acidic bile salts, we analyzed phosphorylation of p47^phox^ at Ser345 and its subcellular localization in CP-A cells treated with BA/A. We found that BA/A cause a rapid phosphorylation of p47^phox^ and its translocation to the cellular membrane fraction (Fig. 5D).

Taken together, our data demonstrates that exposure to BA/A leads to upregulation and activation of NOX proteins, which in turn, causes DNA damage.

### Protein kinase C is involved in the regulation of NADPH oxidases in esophageal cells exposed to acidic bile salts

**Figure 6:**
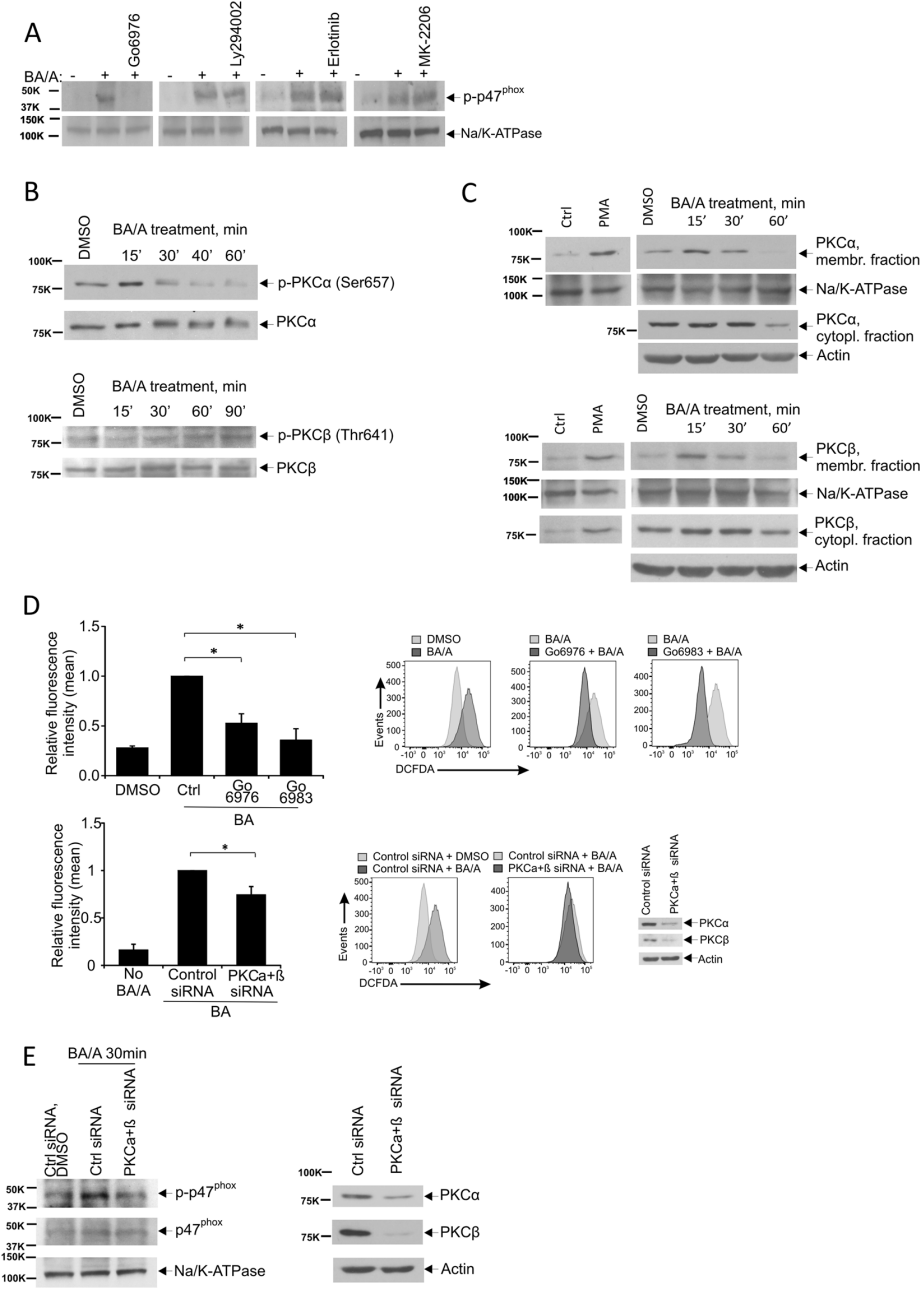
PKCα and PKCβ isoforms are involved in activation of NAD(P)H oxidases and generation of ROS in BA/A-treated cells. (**A**) CP-A cells were pre-treated with the indicated kinase inhibitors for 1 hour, treated with BA/A (100 μM, pH 4.0) and analyzed for phosphorylation of p47^phox^ subunit in the cellular membrane fraction. Only PKC inhibitor Go6976 efficiently blocked phosphorylation of p47^phox^ in BA/A-treated cells. (**B**) Acidic bile salts induce phosphorylation of PKC isoforms. CP-A cells were treated with BA/A and analyzed for PKCα(Ser657) and PKCβ(Thr641) phosphorylation at the indicated time. (**C**) Acidic bile salts induce translocation of PKCα and PKCβ to the cellular membrane. CP-A cells were treated with BA/A and analyzed for the subcellular localization of PKCα and PKCβ. As a positive control, CP-A cells were treated with PMA. (**D**) Inhibition of PKC isoforms decreases levels of ROS after treatment with BA/A. ROS levels were measured by flow cytometry using DCFDA staining in CP-A cells treated with BA/A. Top panels: measurement of ROS levels after inhibition of PKCα and PKCβ with Go6976 and Go6983 compounds. Representative flow cytometry profiles are shown. Bottom panels: measurement of ROS levels after downregulation of PKCα and PKCβ with the specific siRNA. Representative flow cytometry profiles are shown. Downregulation of PKCα and PKCβ is shown on the right bottom panel. Bar graphs show mean values ± S.E. of three independent experiments (*p < 0.05, n = 3). ROS levels in control cells were arbitrarily set to 1. (**E**) Downregulation of PKCα and PKCβ with siRNAs suppresses phosphorylation of p47^phox^ in BA/A-treated cells. CP-A cells, in which PKC isoforms were downregulated, were treated with BA/A for 30 min and analyzed for p47^phox^ phosphorylation in the cellular membrane fraction. Na/K-ATPase was used as a loading control. Right panel shows levels of PKCα and PKCβ after downregulation with siRNA.

To investigate how NOX proteins become activated by BA/A, we took advantage of kinase inhibitors that have been previously shown to affect activity of p47^phox^ in different experimental conditions^20, 24, 25^. The following inhibitors were selected: LY 294002(PI3K), Erlotinib(EGFR), MK-2206 2HCl(AKT), and Go6976(PKC). To assess how these inhibitors affects NOX activation in BA-A treated cells, CP-A cells were treated with the inhibitors (10 µM) and analyzed for phosphorylation of p47^phox^ (Ser345) induced by BA/A. Among tested compounds, only Go6976 was found to be effective in inhibition of p47^phox^ phosphorylation (Fig. 6A). Given that Go6976 selectively inhibits PKCα and PKCβ, it suggests that conventional PKC isoforms are involved in the activation of NOXs^26^. To test this possibility, PKCα and PKCβI proteins were analyzed in CP-A cells treated with BA/A. We found that acidic bile salts strongly activate PKCα as its phosphorylation at serine 657, a phosphorylation site that is important for full activation of PKCα kinase, is increased following exposure to BA/A (Fig. 6B). In the same conditions, a less prominent phosphorylation of PKCβ was found (Fig. 6B). Since translocation of PKC kinases from cytosol to the cellular membranes is an important step for their activation and phosphorylation of downstream substrates, we also tested whether the subcellular localization of PKCα and PKCβ is altered after exposure to BA/A. BA/A treated and control untreated cellular lysates were fractionated and analyzed for subcellular localization of PKC kinases. We found that acidic bile salts induce a rapid translocation of PKCα and PKCβ (15–30 minutes after exposure to BA/A) to the cellular membrane fraction (Fig. 6C).

To elucidate the role of PKC in generation of ROS by BA/A, CP-A cells were pre-treated with PKC inhibitor Go6976, which exhibit selectivity for PKCα and PKCβ, treated with BA/A as discussed above, and then generation of ROS was assesses by flow cytometry. Levels of BA/A-induced ROS were significantly decreased following inhibition of PKCα and PKCβ (Fig. 6D; top panels). We repeated this experiment with another PKC inhibitor Go6983, which also inhibit PKCα and PKCβ, and found that Go6983 is also effective in inhibition of ROS induced by BA/A.

To further confirm the role of PKC, we employed a cocktail of siRNAs that specifically downregulates PKCα and PKCβ isoforms. Similar to chemical PKC inhibitors, downregulation of PKCα and PKCβ led to significant suppression of BA/A-induced ROS (Fig. 6D; bottom panels).

These findings led to the next question: Does PKC regulate phosphorylation of p47^phox^ in BA/A-treated cells? To answer this question, we downregulated PKCα and PKCβ with the siRNAs cocktail and assessed phosphorylation of p47^phox^ after treatment with BA/A. As shown in Fig. 6E, downregulation of PKCα and PKCβ is sufficient to inhibit phosphorylation of p47^phox^ implicating PKC in regulation of NOX enzymes in esophageal cells exposed to acidic bile salts.

## Discussion

Multiple epidemiological studies have demonstrated the essential role of GERD in the development of esophageal adenocarcinoma^27^. Repeated damage of esophageal tissues by acidic gastric juice and bile is in the center of pathological alterations that initiate the tumorigenic process. In this study, we focused on the genotoxic aspect of exposure to acidic bile reflux and the mechanisms of ROS generation by acidic bile salts. As a cellular model, we used non-tumorous esophageal cells isolated from patients with Barrett’s esophagus, a metaplastic lesion that typically arises as a consequence of GERD. Our studies found that DNA damage is induced by reflux in GERD patients. It also happens *in vitro*, when an episode of reflux was recapitulated by a short exposure of BE cells to the mixture of acid and bile salts to which the distal esophagus is commonly exposed during gastroesophageal reflux^12^. These conditions were selected based on previous findings suggesting that both bile and gastric acid contribute to esophageal tumorigenesis^4^. We found that DNA damage is mediated by ROS originated from multiple sources. One is mitochondria, producing an increasing amount of mitochondrial ROS (mtROS) in BA/A treated cells. mtROS can be efficiently inhibited by mitochondrial antioxidant MitoTEMPO. Another one are NAD(P)H oxidases that are activated by BA/A. We identified NOX1 and NOX2 as generators of ROS in BA/A-treated cells. We found that protein levels of NOX1 and NOX2 are transiently increased in BA/A-treated cells. Similarly, increased levels of NOXs were found in GERD patients although with a notable variability. A transient increase of NOXs levels after BA/A exposure suggests that NOX levels may temporarily fluctuate in GERD patients following the dynamics of exposure of esophageal tissues to the refluxate. Our data suggest that NOX proteins are regulated by posttranslational mechanisms since our analyses did not show significant changes in the levels of NOX1 and NOX2 mRNA. Previous studies have demonstrated that NOX levels can be regulated by multiple posttranslational mechanisms such as interaction with different regulatory molecules and posttranslational modifications of the NADPH oxidase complexes (reviewed in ref. 28). It is plausible that these mechanisms might be involved in the regulation of NOX proteins during GERD.

A number of our experiments pointed to the important contribution of NOX1 and NOX2 to DNA damage associated with reflux. Inhibition of NOX1 and NOX2 with the specific siRNAs or inhibitory peptide gp91ds-tat, which interferes with interaction of p47^phox^ and gp91^phox^ subunits, significantly suppressed DNA damage in esophageal cells treated with BA/A. These data are consistent with significant inhibition of ROS and DNA damage by apocynin, a well-characterized NOX inhibitor^29^.

Activation of NOXs in esophageal cells includes changes in subcellular localization of the p47^phox^ subunit, which has previously shown to be central for translocation of other NOX subunits and assembly of the active NOX2 holoenzyme^30^. Although NOX1 interacts with other organizing subunit NoxO1, there are also a number of studies suggesting that p47^phox^ is involved (directly or indirectly) in the regulation of NOX1^21–23^.

We found that BA/A cause rapid phosphorylation of p47^phox^ at serine 345 and its translocation to the cell membrane. PKC kinases, specifically PKCα and PKCβ, are involved in this process. Inhibition of PKC with chemical inhibitors or siRNA efficiently decreased the p47^phox^ phosphorylation. It also decreased production of ROS after treatment with BA/A. Notably, inhibition of PKC had only a partial effect on ROS production, supporting our conclusion that during reflux, ROS originate from multiple sources, such as mitochondria and NOX5-S^6–9^.

The mechanism of PKC activation is currently unclear. It’s plausible that both bile salts and acid contribute to activation of PKC as previous studies have reported that acidic pH and various bile salts can directly stimulate the PKC activity^31–33^. Further investigation is needed to investigate this phenomenon in our experimental model.

Since NOX1 and NOX2 belong to the large NOX/Duox family of ROS-generating enzymes, we cannot exclude that additional members of this family are involved in ROS generation in cells exposed to acidic bile salts. Previous reports have identified NOX5-S to be activated in esophageal cells treated with acid^6–9^. Since the mechanism of activation of NOX5-S does not involve the p47^phox^ subunit, it suggests that NOX1/2 and NOX5-S may play distinct roles in intracellular signaling in esophageal cells exposed to reflux, but both contribute to induction of ROS and DNA damage in the esophagus.

In summary, our data show that exposure of esophageal cells to acidic bile salts induces ROS that, in turn, causes strong DNA damage. The induced ROS originates from mitochondria and NAD(P)H oxidases, NOX1 and NOX2. Both NOXs are upregulated and activated by BA/A. We also found that exposure to BA/A induces PKC, which phosphorylates p47^phox^ subunit, leading to NOX activation. Collectively, our studies support the concept that inhibition of ROS can be a viable strategy for prevention of DNA damage and tumorigenic transformation induced by GERD.

## Methods

### Cell lines

CP-A and BAR-T cells were isolated from Barrett’s epithelium and immortalized with hTERT. CP-A cell line was purchased from ATCC (Manassas, VA) and BAR-T was kindly provided by Dr. Souza (UT Southwestern Medical Center, TX). Cell lines were authenticated and characterized by the suppliers^34^. ATCC uses morphology, karyotyping and PCR-based approaches to confirm the identity of cell lines. Both cell lines were cultured in keratinocyte SFM media supplemented with 40 μg/ml bovine pituitary extract, 1.0 ng/ml epidermal growth factor (Life Technologies, Carlsbad, CA) and 5% fetal bovine serum.

### Treatment with acidic bile salts (BA/A)

Esophageal cells were treated with acidic bile salt cocktail consisting of a 20 μM equimolar mixture of glycocholic, taurocholic, glycodeoxycholic, glycochenodeoxycholic and deoxycholic sodium salts (all reagents were from Sigma-Aldrich, St. Louis, MO); total bile salt concentration was 100 μM. For cell treatment, the bile salt cocktail was diluted in Dulbecco’s modified Eagle’s medium, pH 4.0 (BA/A); pH was adjusted with HCl. CP-A and BAR-T cells were respectively treated with BA/A for 30 min and 5 min and then media were replaced.

### siRNA, antibodies and chemicals

Small interfering RNA (siRNA) were used for downregulation of the following targets: NOX1 (HSS178286, ThermoFisher, Waltham, MA), NOX2 (sc-35503, Santa Cruz Biotechnology, Dallas, TX), PKCα (sc-36243, Santa Cruz Biotechnologies), PKCβ (s11095, ThermoFisher, Waltham, MA). Negative control siRNA with sequence, which does not target any gene product, was from Ambion. Lipofectamine 2000 (ThermoFisher, Waltham, MA) was used for transfection of siRNAs.

The following antibodies were used: NOX2 (polyclonal) and Na/K-ATPase (Clone EP1845Y) from Abcam (Cambridge, MA); p-H2AX (clone 20E3), H2AX (polyclonal),p47phox (polyclonal) and PKCα (polyclonal) from Cell Signaling Technology (Danvers, MA); p-p47phox (polyclonal), and β-actin (clone AC-15) from Sigma-Aldrich; p-PKCα (Ser657, polyclonal), PKCβI (clone E- 3), PKCβII (C18), and p47phox (clone D-10) from Santa Cruz Biotechnology (Dallas, TX); p-PKCβ (Thr641, polyclonal) from Invitrogen (Carlsbad, CA). NOX1 antibodies were from Abcam (polyclonal) and Sigma-Aldrich (polyclonal). They were used for Western blotting and IHC, respectively.

The following chemicals were used: NAC (N-acetylcysteine, Sigma-Aldrich), apocynin (4-hydroxy-3-methoxyacetophenone, Sigma-Aldrich), tempol (4-hydroxy-2,2,6,6-tetramethylpiperydine-1-oxyl, Enzo Biochem, Farmingdale, NY), MitoTEMPO ((2-(2,2,6,6-Tetramethylpiperidin-1-oxyl-4-ylamino)-2-oxoethyl)triphenylphosphonium chloride, Enzo Biochem), Go6976 (5,6,7,13-Tetrahydro-13-methyl-5-oxo-12*H*-indolo[2,3-*a*]pyrrolo[3,4-*c*]carbazole-12-propanenitrile, PKC inhibitor, TOCRIS Bioscience, Bristol, UK), LY 294002 (2-(4-Morpholinyl)-8-phenyl-4H-1-benzopyran-4-one, PI3K inhibitor, Calbiochem, San Diego, CA), Erlotinib (N-(3-ethynylphenyl)-6,7-bis(2-methoxyethoxy)quinazolin-4-amine, EGFR inhibitor, Santa Cruz Biotechnology), MK-2206 2HCl (Akt inhibitor, Selleckchem), Go6983 (3-[1-[3-(Dimethylamino)propyl]-5-methoxy-1*H*-indol-3-yl]-4-(1H-indol-3-yl)-1H-pyrrole-2,5-dione, PKC inhibitor, Selleckchem), PMA (12-O-tetradecanoylphorbol 13-acetate, Sigma-Aldrich). The NOX2 inhibitor peptide gp91ds (CSTRIRRQL) was synthetized by Genscript (Piscataway, NJ).

### RNA extraction and real-time RT-PCR

Total RNA was extracted using the TRIzol reagent (Ambion) according to the manufacturer’s instructions. RNA was reverse transcribed using the High-Capacity cDNA Reverse Transcription Kit according to the manufacturer’s protocol (Applied Biosystems, Foster City, CA). qRT-PCR was performed as described previously^35^ using the iCycler (Bio-Rad, Hercules, CA) with SYBR green dye. Each sample was assayed in duplicate, and data were normalized to the housekeeping gene HPRT (hypoxanthine guanine phosphoribosyl transferase). Primer sequences for HPRT were TTGGAAAGGGTGTTTATTCCTCA (forward) and TCCAGCAGGTCAGCAAAGAA (reverse), for NOX1 TGGTCATGCAGCATTAAACTTT (forward) and AAAACTCATTGTCCCACATTGG (reverse), and for NOX2 CAAGATGCGTGGAAACTACCTAAGAT (forward) and TCCCTGCTCCCACTAACATCA (reverse). The thermal cycling conditions included an initial heat-denaturing step at 95 °C for 3 min, followed by 30 cycles at 95 °C for 10 s and 55 °C for 30 s. Results were calculated using the 2^−ΔΔCT^ method using a reference gene as a normalizer and untreated control as a reference sample.

### Cell fractionation and Western blotting

Cytoplasmic and membrane fractions were generated using the Subcellular Protein Fractionation Kit from ThermoFisher according to the manufacturer’s protocol. Briefly, cells were scraped and spun down after appropriate treatment. The cell pellet was resuspended in the cytoplasmic extraction buffer and kept at 4 °C for 10 min. The cellular extract was centrifuged and the supernatant was collected as a cytoplasmic extract fraction. The pellet was then resuspended in the membrane extraction buffer and kept at 4 °C with intermittent mixing. The extract was centrifuged and the supernatant was collected as a membrane extract fraction. Lysates were analyzed using western blotting as described previously^36^.

### Comet assay

Alkaline comet assay was performed to determine DNA damage induced by BA/A. 3,000–5,000 cells were collected after appropriate treatment, mixed with LM Agarose (Trevigen, Gaithersburg, MD) and allowed to solidify on flare comet slides (Trevigen) at 4 °C. The slides were then immersed in lysis buffer (10 mM Tris, pH 10, 2.5 M NaCl, 100 mM EDTA, 1% Triton X-100, 10% DMSO) for 2 h at 4 °C. Electrophoresis was then performed on slides in alkaline conditions (300 mM NaOH, 1 mM EDTA, pH > 13). The slides were then washed, fixed with 100% cold ethanol, stained with ethidium bromide, and visualized with Olympus BX41 fluorescent microscope (Olympus, Pittsburg, PA). Tail Moment was measure in a minimum of 50–70 cells per treatment using the Open Comet software.

### Reactive oxygen species (ROS) assay

Cellular ROS levels were determined using flow cytometry with 2′, 7′-dichlorofluorescin diacetate (DCFDA) dye (Sigma-Aldrich). Cells were subjected to appropriate treatment, washed with cold PBS and stained with DCFDA dye (10 μM) in serum-free and Phenol Red-free DMEM for 30 min at 37 °C. The cells were then trypsinized, washed 3 times with cold PBS, and stained with 1 µg/mL propidium iodide to exclude dead cells. For flow cytometry analysis, a gate was set to only include single live cells; 10,000 gated cells were analyzed for each treatment. The arithmetic mean fluorescence intensity of DCFDA was obtained using the BD FACSDiva^TM^ software.

### Mitochondrial ROS assay

Mitochondrial ROS was assessed by staining with the mitochondrial ROS indicator, MitoSOX Red (ThermoFisher) that is oxidized by superoxide, and exhibits red fluorescence. Cells were pre-treated with 0.1 µM MitoTEMPO and exposed to BA/A for 1 h. Treated cells were incubated with MitoSOX Red (5 µM) for 30 min and then with Hoechst 33342 (5 μg/mL) for 5 min for staining the nuclei. Images of cells were taken using the Olympus BX41 fluorescent microscope (Olympus). The quantitation of fluorescence intensity was performed with ImageJ. At least 50 cells were analyzed for each treatment.

### Immunohistochemistry

Thirty archival esophageal biopsies collected at Vanderbilt University Medical Center from patients with GERD and BE as well as patients w/o GERD were used for analyses. The use of all human pathology specimens for research was approved by the Institutional Review Board (IRB) of Vanderbilt University Medical Center. Since only de-identified tissues were included in this retrospective study, the IRB has waived the requirements for informed consent. Specimens were histologically verified and selected for immunohistochemical analyses. Immunohistochemical staining was done using the following antibodies: p-H2AX from Millipore, NOX2 from Abcam, and NOX1 from Sigma-Aldrich. The nuclei were counterstained with hematoxylin. Immunohistochemical results were evaluated for intensity and staining frequency, as described previously^37^. The intensity of staining was graded as 0 (negative), 1 (weak), 2 (moderate) or 3 (strong). The frequency was graded according to the percentage of positive cells. Total scores were calculated by multiplying the intensity score by the percentage of positive cells.

### Statistical analysis

Statistical analyses were performed using the Student’s t-test. Results are expressed as averages ± SE, if not specifically indicated. The difference was considered significant if p < 0.05. The Wilcoxon rank sum test was used for statistical evaluation of immunohistochemical staining.

### Data availability statement

All data generated during this study are included in this published article (and its Supplementary Information files). The data can be also obtained from the authors on reasonable request.

## Electronic supplementary material

Supplementary data
