# Supplementary material for: Activation of NADPH oxidases leads to DNA damage in esophageal cells
Source: Sci Rep. 2017 Aug 30;7:9956. doi: 10.1038/s41598-017-09620-4 (PMC5577233; doi:10.1038/s41598-017-09620-4)
Supplement: Supplementary file 1 — Supplementary data [file 41598_2017_9620_MOESM1_ESM.pdf]

Activation of NADPH oxidases leads to DNA damage in esophageal cells

Vikas Bhardwaj[2#], Ravindran Caspa Gokulan[2#], Andela Horvat[2], Liudmila Yermalitskaya[2], Olga Korolkova [2], Kay M. Washington[3], Wael El-Rifai[2, 3], Sergey I. Dikalov[4], Alexander I. Zaika*[1, 2, 3]

Department of Veterans Affairs, Tennessee Valley Healthcare System, Nashville, Tennessee, USA[1], Department of Surgery[2]; Department of Cancer Biology[3]; Division of Clinical Pharmacology[4], Vanderbilt University Medical Center and Vanderbilt-Ingram Cancer Center, Nashville, Tennessee, USA

*Address Correspondence to Alexander Zaika, alex.zaika@vanderbilt.edu.

[#]These authors contributed equally to this work

Surgical Oncology Research, Vanderbilt University Medical Center, 2220 Pierce Ave, 752 PRB, Nashville, TN 37232, Tel. 1-(615) 322-7209, Fax. 1-(615) 322-7852

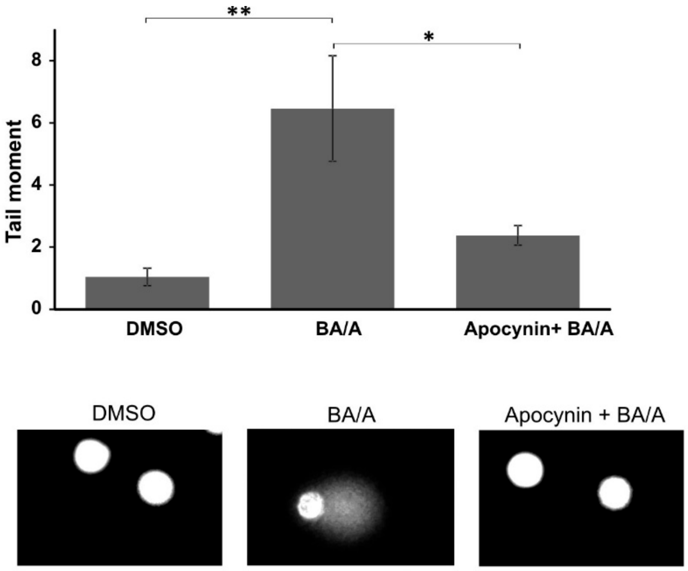

Supplementary Figure 1. Analyses of DNA damage in BAR-T cells after treatment with apocynin. BAR-T cells were pre-treated with apocynin (10 μM) for 1h, exposed to BA/A (100μM, pH 4.0) for 30 minutes and analyzed by comet assay 18 hrs after BA/A treatment (*$p<0.05$, n=3). Representative images of DNA comets are shown. Data are presented as mean ± S.E.

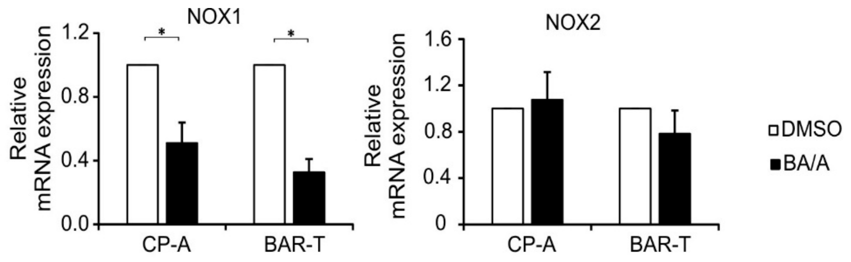

Supplementary Figure 2. Analyses of NOX1 and NOX2 mRNA levels in CP-A and BAR-T cells after treatment with BA/A. mRNA levels in untreated cells were arbitrarily set at 1.
